# Supplementary material for: Activation/Inhibition of Gene Expression Caused by Alcohols: Relationship with the Viscoelastic Property of a DNA Molecule
Source: Polymers (Basel). 2022 Dec 28;15(1):149. doi: 10.3390/polym15010149 (PMC9823369; doi:10.3390/polym15010149)
Supplement: Supplementary file 1 [file polymers-15-00149-s001.zip › polymers-2014053-supplementary.pdf]

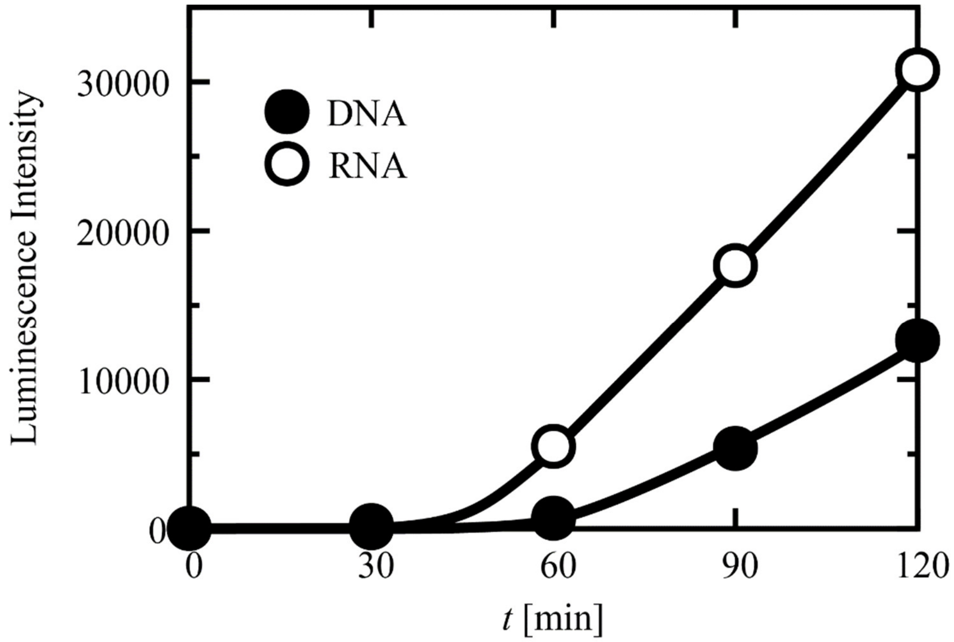

Figure S1. Time-dependent (0–120 minutes) increase in gene expression using the DNA or RNA templates and read as a change in luminescence. The efficiency of gene expression corresponded to the luminescence intensity. The concentrations of DNA and RNA were fixed at 0.3 μM.

Table S1. Detailed experimental data on the efficiency of (a) gene expression (TX-TL) and (b) translation (TL) at different alcohol concentrations of ethanol, 1-propanol and 2-propanol.

(a)

Ethanol

	Luminescence Intensity [RLU]		
Concentration [%]	1st run	2nd run	Average
0	8049	8549	8299
1	21747	21873	21810
2	37904	39140	38522
3	43486	43490	43488
4	22614	22809	22711.5
7	1147	1160	1153.5
10	39	42	40.5

	Luminescence Intensity [RLU]		
Concentration [%]	1st run	2nd run	Average
0	10514	11336	10925
1	30668	32601	31634.5
2	33698	36211	34954.5
3	34279	36464	35371.5
4	19474	19857	19665.5
7	651	678	664.5
10	29	23	26

	Luminescence Intensity [RLU]		
Concentration [%]	1st run	2nd run	Average
0	10425	10455	10440
1	26144	26689	26416.5
2	44316	45626	44971
3	50026	51841	50933.5
4	27687	27102	27394.5
7	1025	1105	1065
10	16	20	18

1-Propanol

	Luminescence Intensity [RLU]		
Concentration [%]	1st run	2nd run	Average
0	110560	120507	115533.5
1	74762	73458	74110
2	31909	31313	31611
3	8666	9076	8871
4	859	953	906
7	24	16	20
10	12	17	14.5

	Luminescence Intensity [RLU]		
Concentration [%]	1st run	2nd run	Average
0	20562	22756	21659
1	12594	14130	13362
2	5892	5947	5919.5
3	1719	1683	1701
4	149	137	143
7	12	22	17
10	21	29	25

	Luminescence Intensity [RLU]		
Concentration [%]	1st run	2nd run	Average
0	17008	19309	18158.5
1	12263	12585	12424
2	5223	5208	5215.5
3	1144	1199	1171.5
4	101	156	128.5
7	30	16	23
10	19	25	22

2-Propanol

	Luminescence Intensity [RLU]		
Concentration [%]	1st run	2nd run	Average
0	8979	9394	9186.5
1	26987	28569	27778
2	38385	40768	39576.5
3	23044	23718	23381
4	13854	13724	13789
7	160	166	163
10	34	22	28

	Luminescence Intensity [RLU]		
Concentration [%]	1st run	2nd run	Average
0	6533	6659	6596
1	20540	20336	20438
2	26817	27049	26933
3	15453	15286	15369.5
4	5710	5859	5784.5
7	102	103	102.5
10	18	28	23

	Luminescence Intensity [RLU]		
Concentration [%]	1st run	2nd run	Average
0	7098	7258	7178
1	15528	15614	15571
2	19966	20706	20336
3	11032	11208	11120
4	5018	5132	5075
7	48	43	45.5
10	20	10	15

(b)

Ethanol

	Luminescence Intensity [RLU]		
Concentration [%]	1st run	2nd run	Average
0	17523	17758	17640.5
1	66358	67697	67027.5
2	92001	94895	93448
3	97418	98969	98193.5
4	73504	74440	73972
7	23580	23494	23537
10	1083	1086	1084.5

	Luminescence Intensity [RLU]		
Concentration [%]	1st run	2nd run	Average
0	16493	16638	16565.5
1	79813	78915	79364
2	136612	141735	139173.5
3	135683	140475	138079
4	106499	107010	106754.5
7	34569	36945	35757
10	633	650	641.5

	Luminescence Intensity [RLU]		
Concentration [%]	1st run	2nd run	Average
0	17996	18695	18345.5
1	71875	73600	72737.5
2	97802	98278	98040
3	106098	109357	107727.5
4	91281	92685	91983
7	16529	21498	19013.5
10	7108	7278	7193

1-Propanol

	Luminescence Intensity [RLU]		
Concentration [%]	1st run	2nd run	Average
0	8420	8935	8677.5
1	14889	15949	15419
2	18249	19370	18809.5
3	13974	14517	14245.5
4	3475	3971	3723
7	65	69	67
10	21	23	22

	Luminescence Intensity [RLU]		
Concentration [%]	1st run	2nd run	Average
0	14123	14506	14314.5
1	24325	25284	24804.5
2	28616	29935	29275.5
3	20877	20978	20927.5
4	4645	5151	4898
7	52	40	46
10	39	27	33

	Luminescence Intensity [RLU]		
Concentration [%]	1st run	2nd run	Average
0	17120	18415	17767.5
1	73687	73502	73594.5
2	98587	97449	98018
3	81650	83825	82737.5
4	65643	65507	65575
7	13444	14371	13907.5
10	1222	1309	1265.5

2-Propanol

	Luminescence Intensity [RLU]		
Concentration [%]	1st run	2nd run	Average
0	9297	10884	10090.5
1	29340	29659	29499.5
2	40324	40776	40550
3	32423	32944	32683.5
4	30672	32089	31380.5
7	6634	6517	6575.5
10	1422	1427	1424.5

	Luminescence Intensity [RLU]		
Concentration [%]	1st run	2nd run	Average
0	18453	19614	19033.5
1	75496	77749	76622.5
2	113465	117746	115605.5
3	99726	99308	99517
4	95122	96102	95612
7	21966	22323	22144.5
10	7528	7421	7474.5

	Luminescence Intensity [RLU]		
Concentration [%]	1st run	2nd run	Average
0	17120	18415	17767.5
1	73687	73502	73594.5
2	98587	97449	98018
3	81650	83825	82737.5
4	65643	65507	65575
7	13444	14371	13907.5
10	1222	1309	1265.5

Additional explanation of Table S1:

We measured the expression level of luciferase by detecting the emission at around 565 nm using a luminometer (MICROTEC Co., Chiba, Japan) after the addition of luciferase substrate (Luciferase Assay Reagent, Promega). Following the manufacturer's instructions, we measured the intensity of luminescence in units of RLU (relative light unit) as an apparent strength of luminescence represented with a luminometer. Then, we evaluated the gene expression efficiency in relative luminescence intensity based on the control condition, which is without adding any alcohols, as shown in Figure 1. Table A1 shows the whole data of measured RLU as raw data of gene expression experiments shown in Figure 1. Both gene expression experiments, TX-TL (Figure 1a) and TL (Figure 1b), were carried out in duplicate runs and independently replicated three times, i.e., we obtained 6 data points for each condition.

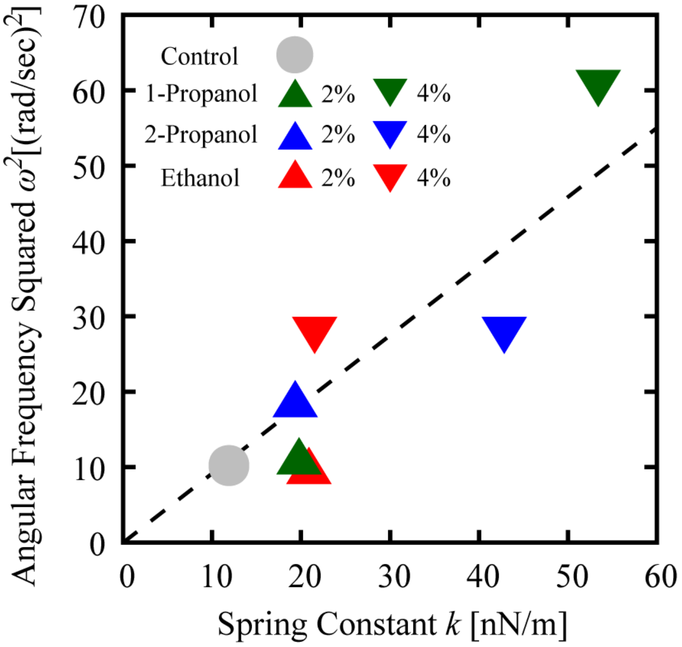

Figure S2. The relationship between k and ω^2. According to the theoretical framework on fluctuation-dissipation, $k \propto \omega^2$ is derived as the fundamental relationship. The dashed line indicates regression estimated by the least-squares method based on the plots for each condition.

Table S2. Viscoelastic parameters evaluated from the analysis of the intrachain fluctuation on single T4 GT7 DNA molecules.

		C(0) [μm²]	Spring constant k [$\times 10^{-9}$Nm^{-1}]	Damping constant γ [sec^{-1}]	Angular frequency ω [rad sec^{-1}]	Damping ratio * ζ
	Control	0.35 ± 0.02	12 ± 0.7	0.9 ± 0.2	3.2 ± 0.2	0.27 ± 0.07
	Ethanol	0.20 ± 0.03	21 ± 3.2	1.1 ± 0.2	3.1 ± 0.2	0.33 ± 0.07
2%	1-Propanol	0.21 ± 0.03	20 ± 2.8	1.3 ± 0.1	3.3 ± 0.3	0.37 ± 0.04
	2-Propanol	0.21 ± 0.03	19 ± 2.8	1.2 ± 0.1	4.3 ± 0.3	0.27 ± 0.03
	Ethanol	0.19 ± 0.02	22 ± 2.3	1.6 ± 0.1	5.3 ± 0.5	0.29 ± 0.03
4%	1-Propanol	0.08 ± 0.01	53 ± 6.6	3.0 ± 0.4	7.8 ± 1.0	0.36 ± 0.07
	2-Propanol	0.10 ± 0.01	43 ± 4.2	1.5 ± 0.2	5.3 ± 0.6	0.27 ± 0.05

$$* \quad \zeta = \frac{\gamma}{\sqrt{\gamma^2 + \omega^2}}$$
